# Latent TB treatment outcomes in patients with chronic liver disease: A retrospective cohort study

**DOI:** 10.1016/j.jctube.2025.100572

**Published:** 2025-11-15

**Authors:** Lilian Tran, Anand Shah, Eugene J. Kim, Sofia Lopiano, Isabella N. Blanchard, Alan Nguyen, David Rutenberg, Berk Madendere, Amee Patrawalla

**Affiliations:** a Rutgers NJMS Department of Medicine, Newark, NJ, USA; b Moffitt Cancer Center, Tampa, FL, USA

**Keywords:** Latent Tuberculosis, Chronic liver disease, Cirrhosis

## Abstract

**Background:**

Chronic liver disease (CLD) increases latent tuberculosis infection (LTBI) reactivation risk, yet treatment is complicated by drug-induced liver injury (DILI) and limited guidance. We compared DILI incidence, treatment completion, and adverse events between cirrhotic and non-cirrhotic patients undergoing LTBI therapy.

**Methods:**

This single-center retrospective study included CLD patients who initiated LTBI treatment (2016–2024). Demographics, comorbidities, treatment regimens, and outcomes were collected by chart review. LTBI was diagnosed by physician documentation or positive Interferon-Gamma Release Assays (IGRAs). CLD etiologies included NAFLD, alcohol-associated liver disease, chronic hepatitis B, chronic hepatitis C, and autoimmune hepatitis. DILI was defined by the U.S DILI Network criteria or treatment alteration.

**Results:**

Among 70 patients with CLD, 24 (34.3 %) had cirrhosis. Overall, 67.1 % completed LTBI treatment with lower rates in cirrhotics (50.0 % vs. 76.1 %, p = 0.035). Adverse effects occurred in 38.6 %, including DILI in 20.0 %. CKD ≥ 3 was associated with more adverse events (p = 0.029). Cirrhosis trended toward higher adverse events (p = 0.0528) but was not predictive of DILI (p = 0.1661). Use of isoniazid for 6 months was associated with a higher DILI risk than rifampin for 4 months (OR 7.21, 95 % CI 1.39–37.31, p = 0.019).

**Conclusions:**

LTBI treatment in patients with CLD is achievable and generally well tolerated despite significant comorbidities. While cirrhosis was associated with lower treatment completion, it did not independently increase DILI risk. Shorter rifamycin-based regimens were safer and better tolerated compared to longer isoniazid-based regimens. Individualized regimen selection and close monitoring are essential to optimizing LTBI treatment outcomes in this high-risk population.

## Highlights

• Chronic liver disease and cirrhosis are known risk factors leading to increased risk of LTBI progressing to active disease.
• LTBI treatment in chronic liver disease is achievable and well-tolerated, especially with shorter rifamycin-based regimens.
• Cirrhosis was associated with lower treatment completion but not with higher DILI risk.
• Prospective studies are needed to refine risk stratification and improve LTBI treatment in chronic liver disease.

## 1 Introduction

Latent Tuberculosis infection (LTBI) affects approximately 13 million individuals in the United States [1,2]. While non-contagious, LTBI carries a 5–10 % lifetime risk of progression to active tuberculosis, with higher risk among immunocompromised individuals [3].

Chronic liver disease (CLD) is a notable risk factor for LTBI progression to active disease due to impaired immune function. Cirrhosis is an immunosuppressive disease characterized by reduced cytokine, bacterial, and endotoxin clearance. Malnutrition, immunosuppressive medications, and alcohol intake further contribute to immune dysfunction. [3,4]. Treatment of LTBI is crucial, as patients with decompensated liver disease experience higher case-fatality rates and have fewer safe treatment options [3]. Rifamycin-based regimens are recommended due to their established efficacy, better tolerability, and higher completion rates due to their shorter durations [1,5]. Despite therapeutic advancements, LTBI management remains complex due to the prolonged duration of therapy, potential for adverse drug events, most notably drug-induced liver injury (DILI), and risk of drug-drug interactions. DILI is implicated in 7 % of adverse drug reactions, 2 % of inpatient jaundice cases, and approximately 30 % of acute liver failure cases [5]. This risk is magnified in patients with pre-existing CLD.

As CLD rates, including nonalcoholic fatty liver disease (NAFLD), alcoholic liver disease (ALD), and cirrhosis continue to rise, treatment of LTBI remains a growing concern [6,7,8]. Current CDC and USPSTF guidelines recommend LTBI screening in individuals at increased risk for exposure or progression to active disease, while the AASLD advises screening as a part of the pre-transplant evaluation due to anticipated immunosuppression [9,10,11]. Yet, guidance on treatment safety and regimen selection in CLD is limited.

Although the American Thoracic Society recommends minimizing hepatotoxic medications, hepatotoxicity remains a significant cause of treatment discontinuation and increased morbidity [1]. Selecting the optimal LTBI regimen is further complicated by limited data in this population. Emerging studies suggest that fluoroquinolone-based regimens may offer a safer alternative with reduced hepatotoxicity compared to traditional isoniazid or rifampin-based therapies [12,13].

Given these complexities, updated clinical data is needed to guide LTBI management in patients with CLD. This study aims to compare the incidence of DILI, treatment completion, and overall adverse effects between cirrhotic and non-cirrhotic patients and to identify other risk factors influencing these outcomes.

## 2 Methods

### 2.1 Study design and population

We performed a single-center retrospective study in a liver transplant center of patients with CLD and LTBI who initiated treatment from the Waymon C. Lattimore Practice in Newark, New Jersey from November 2016-October 2024. The clinic primarily serves an urban, underserved population from TB-endemic and low-income communities identified by the CDC and USPSTF as high-risk for LTBI and progression to active disease [9,10]. All adult patients (≥ 18 years old) diagnosed with LTBI and CLD at treatment initiation were identified through electronic medical record review. We excluded patients who were seen but never started treatment, those with active TB, and those that continued treatment at another facility.

LTBI diagnosis was based on physician documentation and positive Interferon-Gamma Release Assays (IGRA) coupled with the absence of active TB on chest imaging. None had a prior history of treated tuberculosis or known exposure to active TB. We acknowledge that immunosuppressed patients may have false-negative IGRA results despite latent infection and recognize the concept of LTBI-equivalents [14]. However, because our clinic primarily evaluates patients referred for LTBI management who already have positive IGRA results, individuals with negative IGRAs results or potential LTBI-equivalent states were not typically encountered. Given the retrospective design, it was difficult to identify or include such patients, which may introduce referral bias and underrepresent certain high-risk immunosuppressed individuals who could meet LTBI-equivalent criteria despite negative testing, thereby excluding them from our cohort.

CLD diagnoses were also based on physician documentation and included NAFLD, ALD, chronic hepatitis B, chronic hepatitis C, and/or autoimmune hepatitis. Imaging and laboratory results were reviewed for confirmation if available. Cirrhosis diagnosis, including compensated or decompensated, also relied on physician documentation and radiographic findings. Patients with a history of liver transplantation were classified based on their pre-transplant diagnosis. Those who underwent transplant prior to LTBI treatment were still categorized as cirrhotic in this analysis, reflecting their underlying liver disease status at the time of transplantation.

The initial treatment regimen was defined as the first LTBI regimen prescribed, for which the patient received at least one documented dose. All outcomes were assessed relative to this initial regimen. Demographics and comorbidities were extracted from physician documentation at the time of treatment.

We also assessed additional potentially hepatotoxic medications by reviewing medication lists. Only medications with a LiverTox database likelihood score of A or B were included to determine their potential effects on DILI outcomes [15].

### 2.2 Outcomes

DILI was defined by the US DILI Network definition ALT or AST > 5x ULN or ALP > 2x ULN on two consecutive occasions or TBL > 2.5 mg/dL and elevated AST, ALT, or ALP or INR > 1.5 and elevated AST, ALT, or ALP or progressive transaminitis or decompensation prompting treatment alteration by the clinician that did not meet these numerical criteria for ‘DILI.’ We sought to confirm baseline elevation of hepatic enzymes for at least 1 year prior to TB treatment initiation, where data was available.

Other adverse events were defined as any clinical symptoms requiring treatment alteration based on documentation. These were categorized into constitutional (generalized fatigue), gastrointestinal (nausea, vomiting, acid reflux), neurologic (paresthesia, dizziness, headaches), musculoskeletal (muscle aches, joint pains), cardiovascular (prolonged QTC, pericarditis), and dermatologic (rash, itchiness).

Treatment completion and changes in regimen were determined by documentation.

### 2.3 Data analysis and Synthesis

Continuous variables were summarized as means (SD) or medians (IQR) and compared using Student’s *t*-test or Mann–Whitney *U* test; categorical variables were compared using chi-square or Fisher’s exact tests as appropriate. Multivariable logistic regression was performed to identify predictors of DILI, adverse effects, and treatment completion, adjusting for age, sex, BMI, race/ethnicity, alcohol and tobacco use, illicit drug use, and comorbidities including hypertension, diabetes, CKD (stage ≥ 3), heart failure, COPD, and HIV. Statistical analyses were performed using STATA and Microsoft Excel.

## 3 Results

Table 1A summarizes the baseline patient demographics. A total of 157 patients were initially identified. After excluding those who never initiated treatment, had active TB, or continued treatment at another facility, 70 patients were included: 24 (34.3 %) cirrhotic and 46 (65.7 %) non-cirrhotic patients. The mean age was 52.64 years (SD 12.30), with cirrhotic patients significantly older than non-cirrhotics (59.25 vs 49.20 years, p = 0.0004). Cirrhotic patients were also more likely to be male (p = 0.0043)), while BMI and other racial/ethnic categories were similar between groups.

**Table 1A:** Baseline Demographics of Patients with CLD Receiving LTBI Treatment.

**Demographic**	**Total (n = 70)**	**Cirrhotic (n = 24)**	**Non-cirrhotic (n = 46)**	**P-value**
**Age, mean (SD)**	52.64 years old (SD 12.30)	59.25 (SD 9.97)	49.20 (SD 12.06)	0.0004*
**Male**	39 (55.7 %)	19 (79.2 %)	20 (43.5 %)	0.0043 *
**Female**	31 (44.3 %)	5 (20.8 %)	26 (56.5 %)	
**BMI, mean (SD)**	30.95 (SD 7.08)	30.87 (SD 8.65)	30.98 (SD 6.20)	0.956
**Black**	18 (25.7 %)	5 (20.8 %)	13 (28.3 %)	0.4997
**Caucasian**	2 (2.9 %)	1 (4.2 %)	1 (2.2 %)	1.0
**Asian**	6 (8.6 %)	4 (16.7 %)	2	0.1713
**Hispanic**	44 (62.8 %)	14 (58.3 %)	30 (65.2 %)	0.5715

Demographic characteristics of the study cohort (n = 70), stratified by cirrhosis status. Continuous variables are presented as mean (standard deviation − SD) and categorical variables as number (percentage). P-values represent comparisons between cirrhotic and non-cirrhotic groups using t-tests for continuous variables and Fisher’s exact test or chi-square test for categorical variables. * indicates statistical significance at p < 0.05.

Table 1B outlines the different CLD etiologies amongst the two cohorts. NAFLD was the most common among non-cirrhotics (55.7 %), while cirrhotic patients more frequently had alcohol-related disease or viral hepatitis. Among cirrhotics, 70.8 % were decompensated.

**Table 1B:** Underlying Liver Disease Etiology.

**CLD Etiology**	**All CLD (n = 70)**	**Total Cirrhotic (n = 24)**	**Compensated (n = 7)**	**Decompensated (n = 17)**	**Non-Cirrhotic (n = 46)**
NAFLD	39 (55.7 %)	3 (12.5 %)	0	3 (17.6 %)	36 (78.3 %)
ALD	10 (14.3 %)	9 (37.5 %)	3 (42.8 %)	6 (35.3 %)	1 (2.2 %)
Hepatitis C	4 (5.7 %)	4 (16.7 %)	0	4 (23.5 %)	0 (0.00)
Hepatitis B	12 (17.1 %)	4 (16.7 %)	2 (28.6 %)	2 (11.8 %)	8 (17.4 %)
Autoimmune	2 (2.9 %)	1 (4.2 %)	1 (14.3 %)	0	1 (2.2 %)
NAFLD and Hepatitis C	2 (2.9 %)	2 (8.3 %)	0	2 (11.8 %)	0
Alcohol and Hepatitis C	1 (1.4 %)	1 (4.2 %)	1 (14.3 %)	0	0

Distribution of underlying chronic liver disease (CLD) etiologies among all patients (n = 70), stratified by cirrhosis status (compensated vs. decompensated) and presence or absence of cirrhosis. Values are presented as number (percentage) of patients within each subgroup.

NAFLD: non-alcoholic fatty liver disease; ALD: alcoholic liver disease.

Table 2 outlines comorbidities and risk factors. HTN (52.9 %) and alcohol use (48.6 %) were the most common. HTN was significantly more prevalent among cirrhotics compared to non-cirrhotics (70.8 % vs 43.5 %, p = 0.0295). 71.4 % of patients were prescribed at least one additional potentially hepatotoxic medication, with a median count being similar between groups (p = 0.449).

**Table 2:** Patient Co-morbidities and Risk Factors.

**Co-morbidities/Risk Factors**	**Total (n = 70)**	**Cirrhotic (n = 24)**	**Non-cirrhotic (n-46)**	**P-value**
**HTN**	37 (52.9 %)	17 (70.8 %)	20 (43.5 %)	0.0295*
**DM**	27 (38.6 %)	11 (45.8 %)	16 (34.8 %)	0.3673
**CKD3 or >**	6 (8.6 %)	4 (16.7 %)	2 (4.3 %)	0.171
**HF**	2 (2.9 %)	1 (4.2 %)	1 (2.2 %)	1.0
**HLD**	39 (55.7 %)	10 (41.7 %)	29 (63.0 %)	0.0874
**COPD**	6 (8.6 %)	4 (16.7 %)	2 (4.3 %)	0.1713
**HIV**	0	0	0	−
**EtOH**	34 (48.6 %)	14 (58.3 %)	20 (43.5 %)	0.2379
**Tobacco**	18 (25.7 %)	9 (37.5 %)	9 (19.6 %)	0.1032
**Drug use**	3 (4.3 %)	1 (4.2 %)	2 (4.3 %)	1
**# of patients with >/=1 hepatotoxic medication (%)**	50 (71.4 %)	17 (70.83 %)	33 (71.7 %)	0.9365
**Median # hepatotoxic meds**	1 (IQR 0–2)	2 (IQR 0.5–2)	1 (IQR 0–2)	p-value 0.449 (Mann Whitney *U* test)

Baseline comorbidities and risk factors among patients with CLD receiving LTBI treatment, stratified by cirrhosis status. Values are presented as number (percentage) unless otherwise indicated. P-values compare cirrhotic versus non-cirrhotic groups using Fisher’s exact test, chi-square test, *t*-test, or Mann-Whitney *U* test as appropriate. * indicates statistical significance at p < 0.05.

Outcomes are outlined in Table 3A. Overall, 47 patients (67.1 %) completed LTBI treatment, with lower treatment completion among cirrhotics (50.0 % vs 76.1 %, p = 0.0274). Five patients (7.1 %) had unclear completion status and were counted as incomplete. No TB reactivation was observed.

**Table 3A:** Treatment Outcomes.

**Outcomes**	**Total** **n = 70**	**Cirrhotic (n = 24)**	**Non-cirrhotic (n = 46)**	**P value**
**Completed Treatment**	47 (67.1 %)	12 (50.0 %)	35 (76.1 %)	0.0274*
**Death**	1 (1.4 %)	1 (4.2 %)	0	0.343
**Adverse Effects**	27 (38.6 %)	13 (54.2 %)	14 (30.4 %)	0.0528
DILI	14 (20.0 %)	7 (29.2 %)	7 (15.2 %)	0.1661
Constitutional	4 (5.7 %)	2 (8.3 %)	2 (4.3 %)	0.603
Gastrointestinal	9 (12.9 %)	5 (20.8 %)	4 (8.7 %)	0.257
Neurological	6 (8.6 %)	4 (16.7 %)	2 (4.3 %)	0.171
Musculoskeletal	2 (2.9 %)	0	2 (4.3 %)	0.543
Cardiovascular	2 (2.9 %)	2 (8.3 %)	0	0.114
Dermatological	2 (2.9 %)	1(4.2 %)	1 (2.2 %)	1

Treatment outcomes stratified by cirrhosis status, including treatment completion, death, and adverse effects: drug-induced liver injury (DILI), constitutional, gastrointestinal, neurological, musculoskeletal, cardiovascular, and dermatological events. Values are presented as number (percentage) unless otherwise specified. P-values compare cirrhotic and non-cirrhotic groups using Fisher’s exact test or chi-square test. * indicates statistical significance at p < 0.05. Adverse effects occurred in 27 patients (38.6 %), with cirrhotic patients trending toward higher rates compared to non-cirrhotics (54.2 % vs. 30.4 %, p = 0.0528). DILI was the most common adverse event (20.0 %) but was not significantly different between groups (29.2 % cirrhotic vs. 15.2 % non-cirrhotic, p = 0.1661).

Adverse effects including DILI occurred in 27 patients (38.6 %) with a trend towards higher rates among cirrhotic patients compared to non-cirrhotics (54.2 % vs 30.4 %, p = 0.0528). DILI was the most frequent adverse event, affecting 14 patients (20.0 %), with no statistically significant difference between cirrhotics and non-cirrhotics (29.2 % vs 15.2 %, p = 0.1661). Excluding DILI, 13 patients (18.6 %), reported other adverse events, most often gastrointestinal (12.9 %) and neurologic (8.6 %), with no significant differences. Despite experiencing an adverse effect of any kind, 9 patients completed treatment. Two switched from 4R to 6H due to intolerance and ultimately completed treatment after switching; however, because they did not complete the initially prescribed regimen, they were counted as incomplete. In total, 10 patients discontinued treatment due to adverse effects and were subsequently lost to follow-up. One cirrhotic on levofloxacin died (4.2 %) during treatment due to hepatic decompensation, though mortality rates were not statistically different between groups (p = 0.343).

Among the cohort, five patients initiated LTBI treatment within 18 months of receiving a liver transplant and were classified as cirrhotic based on their pre-transplant diagnosis. Three received INH and two were treated with levofloxacin. Regimen selection was individualized based on clinical judgement and potential drug-drug interactions with post-transplant immunosuppressive therapy. Four completed treatment, three experienced non-hepatotoxic adverse effects, but none developed DILI. When excluding these patients from the cirrhosis group, treatment completion among cirrhotics decreased from 50 % to 42.1 %, but the association between cirrhosis and lower completion remained significant (p = 0.0085). For adverse effects, the p-value increased from 0.0528 to 0.0917, reflecting a weaker trend.

To further characterize outcomes within the cirrhotic cohort, we also compared compensated and decompensated patients. After excluding post-transplant cases, DILI incidence (p = 0.603) and overall adverse effects (p = 0.141) did not differ significantly between groups, whereas treatment completion was markedly lower among decompensated cirrhotics (p = 0.0061).

In addition to the five patients who received LTBI treatment after liver transplant, five other patients underwent liver transplantation following LTBI initiation. Only one of these five completed treatment. Interruptions were due to hospitalizations for hepatic encephalopathy, gastrointestinal bleeding, or renal failure. Two patients were instructed to stop therapy due to clinical deterioration or adverse effects and were subsequently lost to follow up.

Treatment regimen distribution is reported in Table 3B. 4R was the most common overall (61.4 %) in both cirrhotics (45.8 %) and non-cirrhotics (69.6 %) followed by levofloxacin in cirrhotics (25 %) and 6H in non-cirrhotics (15.2 %). In multivariable analysis, 6H was associated with greater DILI risk than 4R (OR 7.21, p = 0.019, CI 1.393–37.313). DILI was independently associated with treatment non-completion (OR 0.15, p = 0.012, CI 0.036–0.660), while cirrhosis (p = 0.839) was not independently predictive of DILI after adjustment.

**Table 3B:** Initial Treatment Regimen.

	**All Patients (n = 70)**				**Cirrhosis (n = 24)**				**Non-cirrhotic (n = 46)**			
**Tx Regimen**	**No. Patients (%)**	**Completed Tx**	**AE**	**Death**	**No. Patients (%)**	**Completed Tx**	**AE**	**Death**	**No. Patients (%)**	**Completed Tx**	**AE**	**Death**
4R	43 (61.4)	32 (45.7)	13 (18.6)	0	11 (45.8)	6	5 (20.8)	0	32 (69.6)	26 (56.5)	8 (17.4)	0
3HR	2 (2.9)	1 (1.4)	1 (1.4)	0	0	−	−	−	2 (4.3)	1 (2.2)	1 (2.2)	0
3HP	4 (5.7)	4(5.7)	0	0	0	−	−	−	4 (8.7)	4 (8.7)	0	0
6H	11 (15.7)	5 (7.1)	9 (12.9)	0	4 (14.7)	2 (8.3)	4 (16.7)	0	7 (15.2)	3 (6.5)	5 (10.9)	0
LQ	7 (10.0)	4 (5.7)	2 (2.9)	1 (1.43)	6 (25.0)	3 (12.5)	2 (8.3)	1 (4.2)	1 (2.2)	1 (2.2)	0	0
9H	1 (1.4)	1 (1.4)	1 (1.4)	0	1 (4.2)	1 (4.2)	1 (4.2)	0	0	−	−	−
Rifabutin	2 (2.9)	0	1 (1.4)	0	2 (8.3)	0	1 (4.2)	0	0	−	−	−
Total	70	47 (67.1)	27 (38.6)	1 (1.43)	24	12 (50.0)	13 (54.2)	1 (4.2)	46	35 (76.1)	14 (30.4)	0

Initial LTBI treatment regimens and associated outcomes stratified by cirrhosis status. The table reports the number and percentage of patients treated with each regimen (tx regimen), rates of treatment completion (completed tx), adverse effects (AE), and deaths. 4R was the most commonly used regimen. Patients on 6H demonstrated a higher incidence of DILI (OR 7.21, p = 0.019, CI 1.393–37.313) and lower treatment completion compared to those on 4R. Cirrhotic patients experienced higher rates of adverse events than non-cirrhotic patients.

4R: rifampin 600 mg daily for 4 months; 3HR: rifampin + INH daily for 3 months; 3HP: rifapentine + INH + B6 weekly for 3 months; 6H: INH + B6 for 6 months; LQ: Levofloxacin for 6–9 months; 9H: INH + B6 for 9 months.

Among patients who developed DILI, no demographics or comorbidities were significantly associated with DILI occurrence. Additionally, the use and the number of other potentially hepatotoxic medications with a LiverTox score of A or B was not significantly associated with DILI occurrence [11]. CKD ≥ 3 was significantly associated with a higher risk of adverse effects (p = 0.029) while DM (p = 0.083) demonstrated a trend towards higher risk of adverse events. Cirrhosis was significantly associated with lower completion rates (p = 0.035).

## 4 Discussion

Data on LTBI treatment outcomes in CLD populations remains limited. Our study demonstrates that while cirrhosis is associated with lower treatment completion, it is not independently predictive of DILI. Moreover, treatment completion is achievable despite comorbidities and concurrent hepatoxic medications.

Initiating LTBI treatment in cirrhotic patients remains challenging due to concern for hepatic decompensation and DILI. However, our study suggests that this should not be an absolute barrier to treatment. Cirrhosis was not independently associated with an increased risk of DILI (p = 0.839), with the overall DILI incidence at 20 %, equally distributed between cirrhotic and non-cirrhotic patients. This aligns with findings by Park et al., who observed similar hepatotoxicity rates during active TB treatment regardless of cirrhosis status [16]. Collectively, these data suggest that cirrhosis alone should not preclude LTBI therapy.

Although our subgroup of compensated and decompensated cirrhotics was small, treatment completion was lower among decompensated patients, emphasizing the importance of timing therapy relative to disease severity and transplant evaluation. This observation is consistent with the 2019 American Society of Transplantation guideline recommending LTBI treatment post-transplant when feasible [17].

Regimen selection remains crucial in mitigating hepatotoxicity. In our study, patients receiving 6H had significantly higher odds of DILI (OR 7.21, p = 0.019) and lower treatment completion compared to those on 4R. These findings mirror prior studies showing greater safety and adherence with rifamycin-based regimens compared to INH. In a study by Ronald et al., 4R demonstrated higher treatment completion and lower rates of adverse effects, including hepatotoxicity and death, compared to 9H [18]. Similarly, Edwards et al.’s study described a case series of active TB and liver disease, including cirrhotics and non-cirrhotics. Rates of DILI were high and increased with more hepatotoxic drugs. In cirrhotics, rifampin was more likely to be tolerated than INH [12]. These results underscore the importance of regimen selection, with shorter rifamycin-based regimens appearing safer and better tolerated in patients with CLD.

In contrast Grim et al [19], and Sidhu et al [20], found no significant differences in hepatotoxicity between INH and rifampin in transplant candidates. These differences may reflect the greater disease severity in transplant populations. Our cohort, which may have included patients with less advanced CLD, was more comparable to those in Ronald et al., and Edwards et al.’s studies.

Fluoroquinolones (FQs), particularly levofloxacin, are emerging alternatives for LTBI treatment in cirrhotics and transplant patients given their association with lower hepatotoxicity risk compared to standard regimens. FQs demonstrate good *in vitro* activity against *Mycobacterium tuberculosis* and high treatment completion rates ranging from 83–100 % [13,21]. However, as prophylaxis in liver transplant candidates, levofloxacin has been associated with a high incidence of tenosynovitis, which may limit its utility [22]. In our study, 7 patients were treated with levofloxacin, 4 (57 %) completed therapy without adverse effects, none experienced musculoskeletal complications, and 1 died during treatment. While the sample size is small, our findings align with emerging evidence supporting FQs as a reasonable alternative in high-risk CLD populations.

Transplant recipients are at an even greater risk of developing active TB, with most cases resulting from reactivation of latent disease [23]. Current guidelines recommend INH as first-line therapy for liver transplant recipients, as rifamycins can interact with calcineurin inhibitors and other anti-rejection medications. Levofloxacin may be considered a second-line alternative in the post-transplant setting (21). In a study by Lee et al., 17.5 % of patients discontinued therapy, most commonly due to elevated liver enzymes (36.7 %) (23). In contrast, none of the five post-transplant patients in our study developed DILI, four completed therapy, and one required regimen change to levofloxacin but ultimately completed treatment. However, post-transplant patients have distinct physiology and immunosuppressive regimens that may affect DILI susceptibility compared to those with true cirrhosis. Conversely, patients who initiated treatment before transplant often experienced treatment interruptions due to hepatic decompensation or hospitalization, underscoring the importance of treatment timing.

Nationally, LTBI treatment completion rates range from 20-65 %, often hindered by multifactorial barriers [24]. In our study, treatment completion was significantly lower among cirrhotics than non-cirrhotics (50.0 % vs 76.1 %, p = 0.0274), potentially due to medical and psychosocial factors. Cirrhotic patients are often on complex medication regimens, averaging between 3–10 and cognitive impairments from hepatic encephalopathy can further compromise adherence [25]. Nonetheless, our study demonstrated that 67.14 % of patients — despite high comorbidity burden — successfully completed treatment and 38.57 % did so without experiencing any adverse effects. Therefore, LTBI treatment is feasible even in medically complex populations.

Interestingly, a greater proportion of patients with cirrhosis were prescribed hepatotoxic medications, defined as those with LiverTox likelihood scores of A or B. This included commonly used agents such as statins, proton pump inhibitors, selective serotonin reuptake inhibitors (SSRIs), and ACE inhibitors. These medications are widely used for comorbidities common in this population and are generally safe with appropriate monitoring. Thus, this likely reflects the greater burden of comorbidity and polypharmacy.

CKD stage >/=3 was significantly associated with higher rates of adverse effects (p = 0.029), though most patients still completed therapy. This observation aligns with prior studies showing that even patients with high comorbidity burdens can complete treatment [26]. Our results reinforce the importance of individualized care and close monitoring, particularly those with concomitant renal impairment, but suggest adverse effects do not necessarily preclude successful treatment.

### 4.1 Limitations

This single-center, retrospective study relied on manual chart review, which may have led to incomplete or inconsistent reporting of adverse events and treatment completion. The small sample size limited the power to detect differences in DILI and comorbidity-related risks.

Classification of post-transplant patients as cirrhotic may not fully reflect hepatic function at the time of therapy, as post-transplant patients have distinct physiology and immunosuppressive regimens that can alter susceptibility to DILI. While MELD data were incomplete, available scores were comparable to true cirrhotics. Reclassification slightly reduced completion rates but did not change overall significance, highlighting the importance of accurate classification and complete liver function data in retrospective studies.

Similarly, although simple steatosis without fibrosis may represent a less advanced form, NAFLD is still characterized as part of the chronic liver disease spectrum, given its potential for progression and associated metabolic injury. However, because we did not stratify patients by fibrosis or disease stage, differences in treatment tolerance or DILI risk across disease severities may have been obscured [27].

Finally, as a U.S.-based tertiary center with close follow-up, our findings may not be generalizable to lower-resourced or high-TB-burden settings. Nonetheless, this study offers novel clinical observations and quantifiable assessments of LTBI treatment outcomes in CLD patients.

### 4.2 Future studies

Future research should include prospective cohort studies with standardized monitoring of DILI, adverse events, and treatment completion. Larger multicenter studies will allow stratification by liver disease severity using scoring systems (e.g., MELD or Child-Pugh). Prospective documentation of DILI onset time, exact LTBI therapy duration, and treatment interruptions would offer more granular insights into tolerability. Randomized controlled trials comparing rifamycin-based regimens to alternative, potentially safer therapies like FQ-based regimens, are warranted for this high-risk population. Additionally, studies should explore the impact of concurrent hepatotoxic medications on treatment tolerability and the safety and efficacy of LTBI treatment in liver transplant recipients.

## 5 Conclusion

Our single-center retrospective study demonstrates that LTBI treatment is feasible and generally well-tolerated in patients with CLD and cirrhosis, despite their significant comorbidities, with close following up in a multidisciplinary setting. Shorter rifamycin-based regimens appear safer and better tolerated than longer isoniazid-based regimens, emphasizing the need for careful regimen selection and monitoring. Importantly, CLD alone shouldn't preclude LTBI treatment or the use of other necessary hepatotoxic medications for comorbidities. High treatment success rates are achievable, but further prospective studies are needed to refine risk stratification and optimize treatment strategies and monitoring for this population.

## Ethical Statement

This study was conducted in accordance with the ethical standards of the institutional and national research committee and with the 1964 Helsinki Declaration and its later amendments. The protocol was reviewed and approved by the Rutgers University Institutional Review Board (IRB #Pro2024000496). Given the retrospective nature of the study, informed consent was waived.

## CRediT authorship contribution statement

**Lilian Tran:** Writing – review & editing, Writing – original draft, Visualization, Validation, Project administration, Methodology, Investigation, Formal analysis, Data curation, Conceptualization. **Anand Shah:** Formal analysis, Data curation. **Eugene J. Kim:** Investigation, Data curation. **Sofia Lopiano:** Investigation, Data curation. **Isabella N. Blanchard:** Investigation, Data curation. **Alan Nguyen:** Investigation, Data curation. **David Rutenberg:** Writing – review & editing, Project administration, Methodology, Conceptualization. **Berk Madendere:** Writing – review & editing. **Amee Patrawalla:** Writing – review & editing, Supervision, Resources, Data curation, Conceptualization.

## Funding

This research did not receive any specific grant from funding agencies in the public, commercial, or not-for-profit sectors.

## Declaration of competing interest

The authors declare that they have no known competing financial interests or personal relationships that could have appeared to influence the work reported in this paper.
